# Supplementary figures and images for: Differential immunomodulation of T-cells by immunoglobulin replacement therapy in primary and secondary antibody deficiency
Source: PLoS One. 2019 Oct 15;14(10):e0223861. doi: 10.1371/journal.pone.0223861 (PMC6793872; doi:10.1371/journal.pone.0223861)

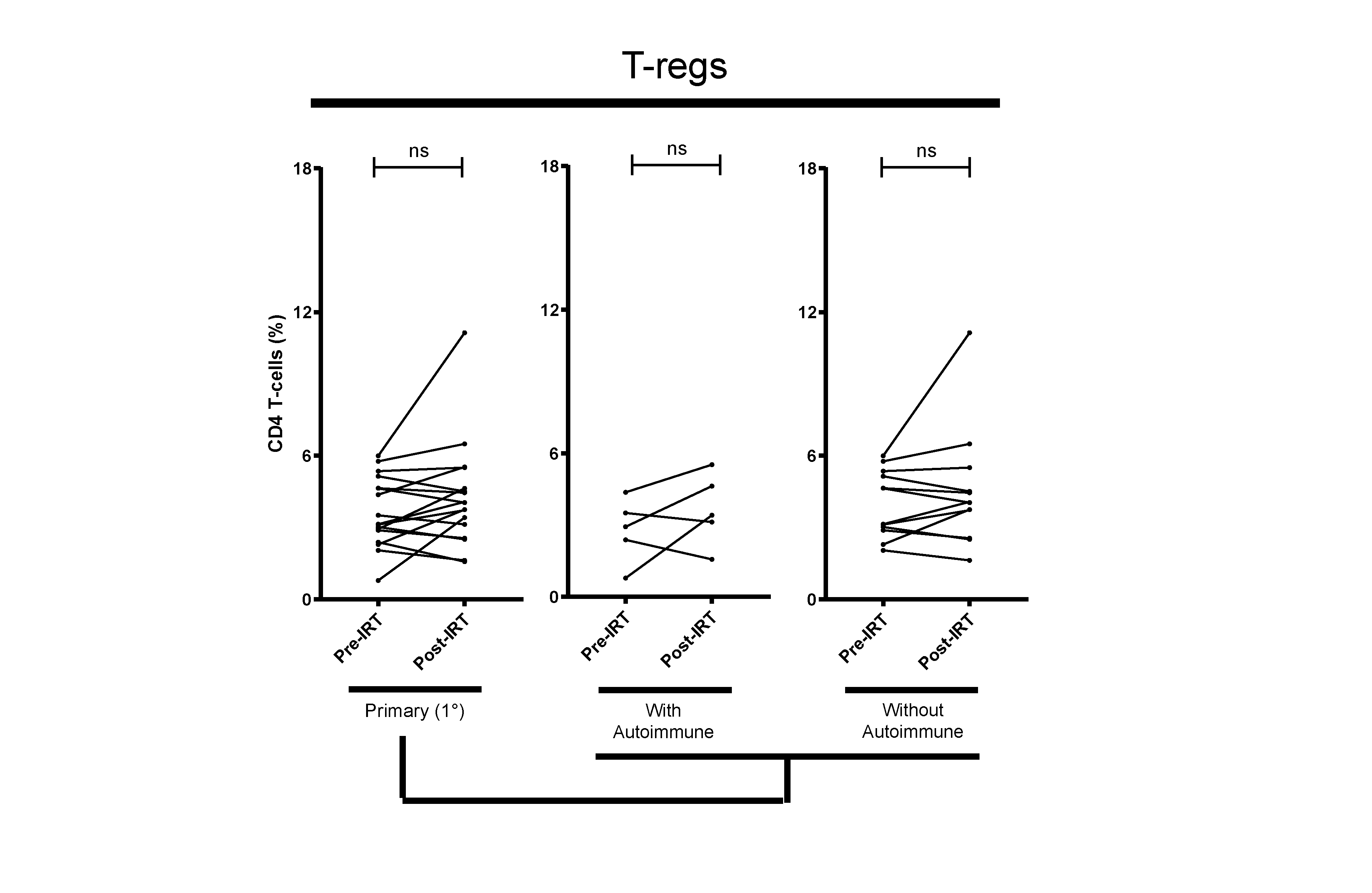

Supplement: S1 Fig — Thawed cryopreserved PAD patient PBMCs were stained for CD3, CD4, CD25, CD127, and FOXP3 for Tregs (CD3+CD4+CD25hiCD127loFOXP3+). PAD patients (left panel) were subdivided based on either presence (center panel) or absence (right panel) of autoimmune disease. For each group, the proportion of Tregs among CD4+ T-cells were compared between pre- and post-IRT. Ns denotes not significant (p > 0.05). P-values were determined by Wilcoxon matched-pairs signed rank test. (TIFF) [file pone.0223861.s001.tiff]
